# The Virulence Effect of CpxRA in *Citrobacter rodentium* Is Independent of the Auxiliary Proteins NlpE and CpxP

**DOI:** 10.3389/fcimb.2018.00320

**Published:** 2018-09-18

**Authors:** Natalia Giannakopoulou, Nilmini Mendis, Lei Zhu, Samantha Gruenheid, Sebastien P. Faucher, Hervé Le Moual

**Affiliations:** ^1^Department of Microbiology and Immunology, McGill University, Montreal, QC, Canada; ^2^Department of Natural Resource Sciences, McGill University, Sainte-Anne-de-Bellevue, QC, Canada

**Keywords:** *Citrobacter rodentium*, virulence, two component systems, intestinal infection, CpxRA, bacterial gene expression

## Abstract

*Citrobacter rodentium* is a murine pathogen used to model the intestinal infection caused by Enteropathogenic and Enterohemorrhagic *Escherichia coli* (EPEC and EHEC), two diarrheal pathogens responsible for morbidity and mortality in developing and developed countries, respectively. During infection, these bacteria must sense and adapt to the gut environment of the host. In order to adapt to changing environmental cues and modulate expression of specific genes, bacteria can use two-component signal transduction systems (TCS). We have shown that the deletion of the Cpx TCS in *C. rodentium* leads to a marked attenuation in virulence in C3H/HeJ mice. In *E. coli*, the Cpx TCS is reportedly activated in response to signals from the outer-membrane lipoprotein NlpE. We therefore investigated the role of NlpE in *C. rodentium* virulence. We also assessed the role of the reported negative regulator of CpxRA, CpxP. We found that as opposed to the Δ*cpxRA* strain, neither the Δ*nlpE*, Δ*cpxP* nor the Δ*nlpE*Δ*cpxP* strains were significantly attenuated, and had similar *in vivo* localization to wild-type *C. rodentium*. The *in vitro* adherence of the Cpx auxiliary protein mutants, Δ*nlpE*, Δ*cpxP*, Δ*nlpE*Δ*cpxP*, was comparable to wild-type *C. rodentium*, whereas the Δ*cpxRA* strain showed significantly decreased adherence. To further elucidate the mechanisms behind the contrasting virulence phenotypes, we performed microarrays in order to define the regulon of the Cpx TCS. We detected 393 genes differentially regulated in the Δ*cpxRA* strain. The gene expression profile of the Δ*nlpE* strain is strikingly different than the profile of Δ*cpxRA* with regards to the genes activated by CpxRA. Further, there is no clear inverse correlation in the expression pattern of the Δ*cpxP* strain in comparison to Δ*cpxRA*. Taken together, these data suggest that in these conditions, CpxRA activates gene expression in a largely NlpE- and CpxP-independent manner. Compared to wildtype, 161 genes were downregulated in the Δ*cpxRA* strain, while being upregulated or unchanged in the Cpx auxiliary protein deletion strains. This group of genes, which we hypothesize may contribute to the loss of virulence of Δ*cpxRA*, includes T6SS components, *ompF*, the regulator for colanic acid synthesis, and several genes involved in maltose metabolism.

## Introduction

Enteropathogenic and Enterohemorrhagic *Escherichia coli* (EPEC and EHEC) are Gram-negative food-borne diarrheal pathogens, transmitted through the fecal-oral route (Mundy et al., 2005). They are responsible for high morbidity and mortality in both the developed and developing world. In the case of EHEC, disease can progress to hemorrhagic colitis and hemolytic uremic syndrome due to the production of the Shiga toxin, which is lacking in EPEC (Mundy et al., 2005). The related murine pathogen *Citrobacter rodentium* is a natural mouse pathogen, first isolated in Japan and the United States of America as the etiologic agent of transmissible murine colonic hyperplasia in mouse colonies (Muto et al., 1969; Barthold et al., 1976). *C. rodentium* is a widely used model to study EPEC and EHEC due to their pathological similarity and difficulty in infecting mice with the human pathogens (Barthold et al., 1976; Schauer and Falkow, 1993). EHEC, EPEC, and *C. rodentium* are members of a group of pathogens known for their ability to form attaching and effacing lesions (A/E lesions) during infection (Mundy et al., 2005). Specifically, the bacteria attach to intestinal epithelial cells, efface the microvillar architecture, and form actin-rich pedestals beneath the adherent bacteria (Moon et al., 1983; Mundy et al., 2005).

To survive during a host infection, bacteria must be able to sense the surrounding environment and adapt their gene expression accordingly. One of the ways in which bacteria sense the environment is through the use of two-component signal transduction systems (TCSs). TCSs are typically composed of a membrane-bound sensor kinase and a cytoplasmic response regulator. Following activation by a stimulus, the sensor kinase will become auto-phosphorylated on a histidine residue in the cytoplasmic domain (Mascher et al., 2006). The phosphate is then transferred to a conserved aspartate residue on a cytoplasmic response regulator, which will carry out a specific transcriptional response, either upregulating or downregulating target genes, generally by binding directly to the cognate DNA sequences of the target gene (Raivio and Silhavy, 1997; Mascher et al., 2006).

Previous work by our group and others has uncovered several TCSs involved in the regulation of virulence properties of *C. rodentium*. Specifically, the inactivation of RstAB, UhpAB, ZraRS, RcsBC, and ArcAB TCSs leads to delayed mortality in susceptible C3H/HeJ mice (Thomassin et al., 2017). The CpxRA TCS deletion strain has the most striking effect, leading to 100% survival of susceptible mice (Thomassin et al., 2015). The QseBC and QseEF TCSs, which respond to epinephrine and norepinephrine, are also important for the virulence of *C. rodentium* (Moreira et al., 2016).

The CpxRA TCS is one of the key envelope stress responses in Gram-negative bacteria. It is activated by a multitude of signals, including misfolded proteins (Snyder and Silhavy, 1995), alkaline pH (Danese and Silhavy, 1998), changes in membrane lipid composition (Mileykovskaya and Dowhan, 1997), high osmolarity (Prigent-Combaret et al., 2001; Jubelin et al., 2005; Bury-Mone et al., 2009), and attachment to abiotic surfaces (Otto and Silhavy, 2002). The CpxRA TCS is composed of the inner membrane-bound histidine kinase CpxA, and the cytoplasmic response regulator CpxR. Upon sensing an external stimulus, CpxA becomes auto-phosphorylated, and transfers the phosphate group to CpxR.

In addition to our work in *C. rodentium*, the Cpx TCS has been implicated in virulence modulation in other bacteria. In *Haemophilus ducreyi*, deletion of CpxA leads to the bacterium's inability to infect humans by decreasing its serum resistance (Spinola et al., 2010). In *Legionella pneumophila*, CpxRA contributes to the bacterium's ability to replicate in protozoa and controls the expression of a multitude of virulence factors (Gal-Mor and Segal, 2003; Tanner et al., 2016). In *Salmonella enterica* serovar Typhimurium, impaired CpxRA function leads to the inability of the bacteria to replicate in mice, as well as to decreased adherence and invasion of eukaryotic cells (Humphreys et al., 2004). Further, CpxRA is a positive regulator of the virulence factor *virF* of *Shigella sonnei* (Nakayama and Watanabe, 1998). In EHEC, high levels of CpxR are known to act negatively on critical components of EHEC virulence: the Locus of Enterocyte Effacement (LEE) and its associated Type 3 Secretion System (T3SS) (De la Cruz et al., 2016). CpxR represses *ler* and *lon*, two known positive regulators of the LEE, and negatively affects EspABD, the translocators of the T3SS (De la Cruz et al., 2016). However, our previous work in *C. rodentium* did not uncover a striking defect in T3SS activity in the absence of CpxRA (Thomassin et al., 2017). In EPEC, CpxRA has been implicated in the regulation of the Bundle Forming Pilus (BFP), an important adherence factor that is not present in *C. rodentium* (Vogt et al., 2010).

In *E. coli*, the Cpx system is regulated by a periplasmic auxiliary protein, CpxP. Its overexpression dampens the Cpx response through a negative feedback loop, as *cpxP* is one of the most highly regulated genes by the Cpx TCS (Raivio et al., 1999). In contrast, the loss of *cpxP* results in modest upregulation of the Cpx pathway, without making the system blind to inducing cues (Raivio et al., 1999). Upstream of CpxA, the outer membrane-anchored lipoprotein NlpE acts as an activator of the response (Snyder et al., 1995). NlpE-dependent activation of Cpx occurs to suppress toxicity of misfolded proteins, as well as in response to adherence to abiotic surfaces (Snyder et al., 1995; Otto and Silhavy, 2002; Shimizu et al., 2016). There exist some NlpE-independent cues for Cpx activation, such as alkaline pH (Danese and Silhavy, 1998; DiGiuseppe and Silhavy, 2003) and drugs targeting peptidoglycan synthesis (Delhaye et al., 2016). However, there also exist numerous cues in which the role of NlpE is entirely uncharacterized (Laloux and Collet, 2017). The involvement of NlpE and CpxP in *C. rodentium* virulence remains unclear.

In order to gain further insight into the cause of the virulence defect associated with the loss of CpxRA in *C. rodentium*, we characterized the role of the putative upstream activator of the Cpx pathway, NlpE, as well as the role of the most prominent auxiliary protein, CpxP. NlpE has been previously implicated in the activation of Cpx in multiple contexts in *E. coli* and EHEC. However, NlpE activation of Cpx *in vivo* remains uncharacterized. We generated chromosomal deletions of *nlpE, cpxP*, and a double mutant of both genes in *C. rodentium*, and investigated the role of each gene during infection of susceptible C3H/HeJ mice. We found that the effect of CpxRA on *C. rodentium* virulence is NlpE- and CpxP-independent. We further characterized the regulon of CpxRA, NlpE, and CpxP using microarrays, in order to uncover differentially regulated genes, and to provide further insight into the differential effects of these proteins. We found a large number of Cpx target genes that are regulated independently of NlpE and CpxP.

## Materials and methods

### Bacterial strains, plasmids, and growth conditions

All strains and plasmids used in this study are listed in Table S1. Bacterial strains were routinely cultured at 220 rpm at 37°C in Luria Bertani (LB) broth [1% [wt/vol] tryptone, 0.5% [wt/vol] yeast extract, 1% [wt/vol] NaCl]. Subculturing, when needed, was done in Dulbecco's Modified Eagle media (DMEM; Wisent). When appropriate, LB was supplemented with chloramphenicol (Cm; 30 μg/ml) or diaminopimelic acid (DAP; 50 μg/ml). This study was carried out in accordance with the McGill biosafety guidelines and regulations, under biosafety permit number B-07706.

### Construction of deletion strains–*sacB* gene-based allelic exchange

The *C. rodentium* deletion strains were generated by *sacB* gene-based allelic exchange, as described previously (Donnenberg and Kaper, 1991). All primers used in this study are listed in Table S2. Briefly, genomic DNA of *C. rodentium* was used as a template to amplify the upstream and downstream sequences of a target gene (primers 1 and 2, and 3 and 4, respectively). Each segment was digested using XbaI, XhoI, or KpnI, as appropriate (New England Biolabs). Following digestion, the segments were purified and ligated using T4 DNA ligase (Thermo Scientific). Next, the ligated product was PCR-amplified with iProof High-Fidelity DNA Polymerase (Biorad), using primers 1 and 4. The amplified segment was further digested using the appropriate enzymes, and was then ligated into pRE112 plasmid which had been digested with XbaI and KpnI (New England Biolabs). The resulting suicide vector plasmid was transformed into CaCl_2_ chemically-competent *E. coli* χ7213 (Hanahan et al., 1991). The *E. coli* χ7213 strain was used as a donor strain in order to conjugate the suicide vectors into wild-type *C. rodentium*, as previously described (Donnenberg and Kaper, 1991). Briefly, 25 μl of an overnight culture of transformed *E. coli* χ7213 and 25 μl of an overnight culture of *C. rodentium* were combined on the surface of an LB-DAP plate for 1 h at 37°C. The conjugation product was plated on LB agar plates supplemented with Cm (30 μg/ml). Colonies that were Cm-resistant were then plated on peptone agar containing 5% sucrose and incubated at 16–18°C for several days in order to isolate colonies that were sucrose resistant. The sucrose-resistant colonies were also screened for Cm sensitivity. Gene deletion was verified by PCR and sequencing of gDNA (Genome Quebec) using primers 1 and 4.

### *In vivo Citrobacter rodentium* infections

This study was carried out in accordance with the recommendations of the Canadian Council on Animal Care. The protocol was approved by the McGill University Animal Care Committee. Female C3H/HeJ mice were purchased from Jackson Laboratories and maintained in a specific-pathogen-free facility at McGill University. Wild-type or mutant *C. rodentium* DBS100 strains were grown overnight in 3 ml LB broth, 220 rpm, at 37°C. Four- to five-week old female mice were orally gavaged with 100 μl of overnight culture, containing 2–3 × 10^8^ CFU. The infectious dose was verified by plating of serial dilutions of the inoculum on MacConkey agar (Difco). For survival experiments, the mice were monitored daily for 30 days, and were euthanized if any of the following clinical endpoints were met: 20% body weight loss, hunching and shaking, inactivity, or body condition score of <2 (Ullman-Culleré and Foltz, 1999). For fluorescent microscopy, the mice were euthanized on day 9 post infection. To detect *C. rodentium* colonization at days 3, 6, 9 post infection, fecal pellets or the terminal centimeter of the colon were homogenized in 1 ml PBS, serially diluted and plated on MacConkey agar (Difco). *C. rodentium* was identified by its distinctive colony morphology. Plates with colonies between 30 and 300 were enumerated. In the case of low bacterial loads, with undiluted plate counts below 30, this plate was enumerated.

### Adherence assays

*In vitro* adherence assays of *C. rodentium* were performed as previously described (Sit et al., 2015). Briefly, HeLa cells were cultured in DMEM with 10% heat-inactivated fetal bovine serum (FBS), and seeded at 5.0 × 10^4^ per well on glass coverslips in a 24-well plate. Overnight bacterial cultures were grown in 3 ml LB, 220 rpm, at 37°C. Prior to infection, HeLa cells were incubated for 30 min in 1 ml of DMEM (Wisent), supplemented with 2% heat-inactivated FBS (Seradigm). Cells were infected at a starting MOI of 1:100 for 8 h at 37°C, 5% CO_2_. Coverslips were washed 3 times in PBS with calcium and magnesium (Wisent) to remove non-adherent bacteria, and then fixed in 2.5% paraformaldehyde (Thermo) for 15 min. Following fixation, samples were permeabilized in 0.1% Triton X-100 (BioShop) in PBS, and blocked overnight in 2% bovine serum albumin (Sigma) with 0.1% Triton X-100 in PBS. Samples were then stained with a rabbit anti-*Citrobacter* LPS antibody (Mast Group), followed by Alexa 488-conjugated anti-rabbit secondary antibody (Invitrogen) and 4',6-diamidino-2-phenylindole (DAPI; Sigma). Coverslips were mounted in Prolong Gold (Invitrogen) and imaged on a Zeiss Axiovert 200M microscope with a Zeiss Axiocam monochrome camera. Ten random fields of view were assessed per sample, and the total number of bacteria and cells were enumerated using Fiji software (Schindelin et al., 2012).

### Fluorescence microscopy

C3H/HeJ mice were infected with strains of *C. rodentium* as described above. Mice were euthanized on day 9 post infection, and the third most distal centimeter of the colon was fixed in 10% neutral buffered formalin. The tissue was paraffin-embedded and cut into 4 μm sections. The slides were deparafinized in xylene twice for 5 min, followed by rehydration in a gradient of 100% ethanol twice for 5 min, 95% ethanol for 5 min, 70% ethanol for 5 min, and dH_2_O for 5 min. The samples were then boiled in 1.8 mM citric acid and 8.2 mM sodium citrate in dH_2_O for 10 min, for antigen retrieval. The slides were left to cool down at RT for 10 min in the buffer, and were subsequently washed with PBS containing 0.2% Tween 20. Further, the samples were blocked in PBS containing 0.2% Tween 20, 10% FBS, and 3% BSA for 1 h at 37°C. The samples were stained with anti-*Citrobacter* LPS rabbit polyclonal antibody in PBS containing 0.2% Tween 20 and 3% BSA at 4°C for 3 h. Following the primary antibody, the samples were incubated with anti-rabbit Alexa 488 secondary antibody and DAPI in PBS containing 0.2% Tween 20 and 3% BSA at 37°C for 1 h. Finally, the samples were mounted in Prolong Gold, and imaged on a Zeiss Axiovert 200M microscope with a Zeiss Axiocam monochrome camera. Images were assembled using Fiji (Schindelin et al., 2012).

### RNA extraction

Bacteria were cultured overnight in LB broth, 220 rpm, at 37°C. Strains were subcultured 1:50 in 50 ml DMEM, at 220 rpm and 37°C, until an OD_600_ of 0.5–0.6. Cells were pelleted at 4°C. Pellets were resuspended in 1 ml of TRIzol reagent (Life Technologies) and stored at −20°C until further extraction. Samples were thawed at RT, and 200 μl of chloroform was added, followed by 15 s of vigorous shaking. The samples were then incubated at RT for 2–3 min. Contents were transferred to Phase-lock Heavy Gel tubes (Quantabio) and centrifuged at 12,000 × g for 15 min at RT. Following centrifugation, the aqueous layer (about 600 μl) was transferred to a fresh microcentrifuge tube, followed by the addition of 600 μl of isopropanol and 1 μl glycogen (Ambion). The contents were mixed by gently inverting the tube, and were incubated at RT for 10 min. The samples were centrifuged at maximum speed at 4°C for 10 min, and while working on ice, the supernatant was removed. The pellet was washed with 1 ml ice-cold ethanol, and centrifuged at maximum speed for 10 min at 4°C in a microcentrifuge. The supernatant was removed, the pellets were air-dried, and resuspended in 40 μl of RNase-free dH_2_O. Further, RNA samples were treated with Turbo DNase (Ambion) for 30 min. Samples were evaluated by NanoDrop to ensure quality and quantity, and by PCR, using 16S rDNA primers, to ensure no DNA contamination.

### Microarrays

#### cDNA and labeling

RNA was extracted and purified as described above. Labeling was done as described previously (Faucher and Shuman, 2013). Briefly, 15 μg of RNA was reverse transcribed to cDNA with the addition of random hexamers, Superscript II reverse transcriptase (Life Sciences) and a mix of dATP, dTTP, dCTP, dGTP (NEB), and aminoallyl dUTP (Sigma). The cDNA was then labeled using Alexa Fluor 647, whereas genomic DNA (extracted from wild-type *C. rodentium*) was labeled using Alexa Fluor 546 (Invitrogen), as described previously (Faucher and Shuman, 2013).

### Microarray hybridization

The microarray slides were custom-made using photolithography by MYcroarray, with 3 probes of 45 nucleotides each, per gene, against the *C. rodentium* DBS100 genome (Lenz et al., 2015). The labeled cDNA and gDNA were hybridized onto the microarray slide as described previously (Faucher and Shuman, 2013). The microarrays were scanned using an InnoScan microarray scanner (Innopsys), and the data was analyzed using Mapix software. The samples were normalized to wild-type, and the genes with a log_2_ ratio of mutant/wild-type >1 or <-1, and *p* < 0.05 were considered differentially expressed and significant. The microarray data are available from Gene Expression Omnibus, https://www.ncbi.nlm.nih.gov/geo/, accession number GSE114699.

## Results

### Susceptible mice do not survive infection with *Citrobacter rodentium* mutants of the Cpx TCS auxiliary proteins NlpE and CpxP

**Figure 1:**
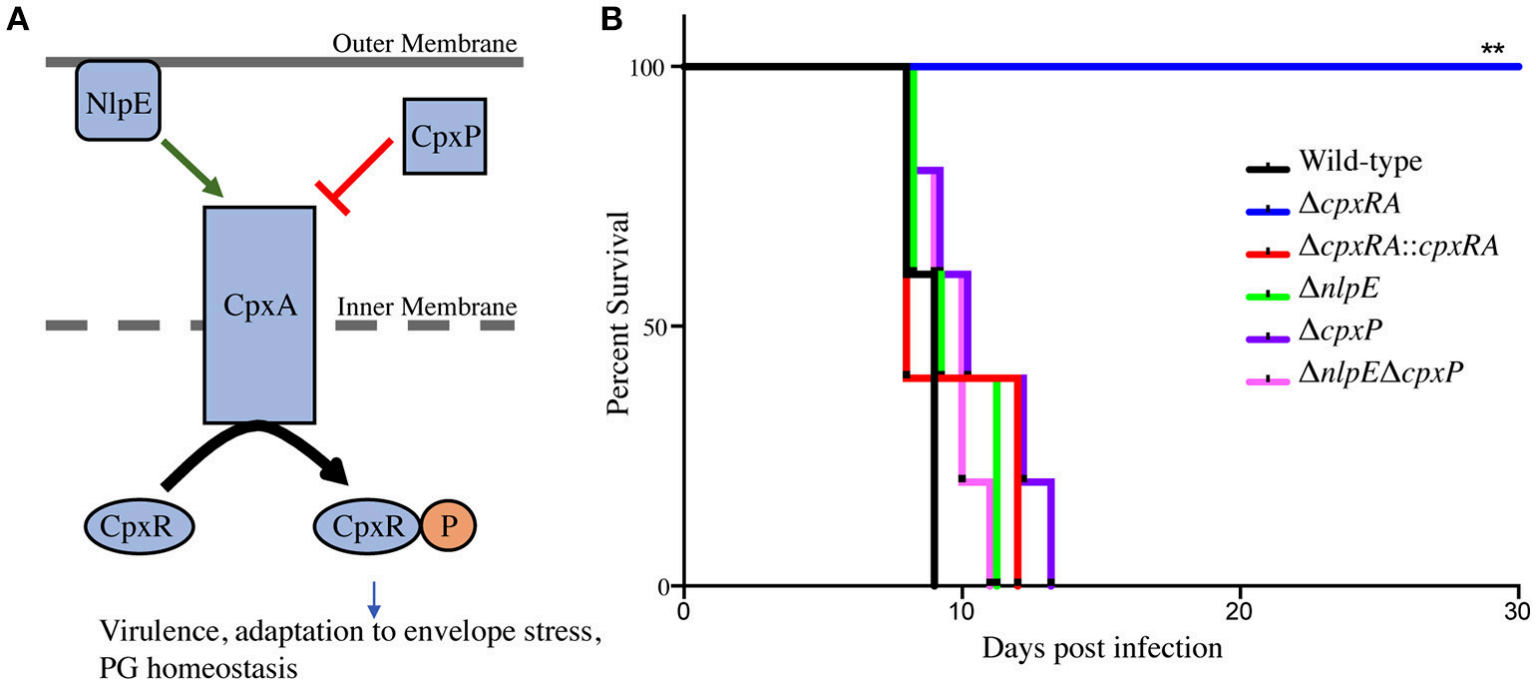
Survival of susceptible mice after infection with Cpx TCS mutant strains of *Citrobacter rodentium*. **(A)** TCSs are used by bacteria in order to sense environmental stress. The membrane bound histidine kinase (CpxA) is activated by an external signal and propagates a cascade of phosphorylation leading to a transcriptional response by the cytoplasmic response regulator (CpxR). The Cpx TCS has a reported upstream outer membrane sensor, NlpE, and a periplasmic inhibitor, CpxP. **(B)** Female C3H/HeJ mice were infected by oral gavage with 2–3 × 10^8^ colony forming units of wild-type *C. rodentium*, or a TCS mutant strain. Survival was monitored for 30 days post infection. The log-rank (Mantel-Cox) method was used to determine statistical significance. (PG - peptidoglycan), (**P < 0.01, *n* = 5).

**Figure 2:**
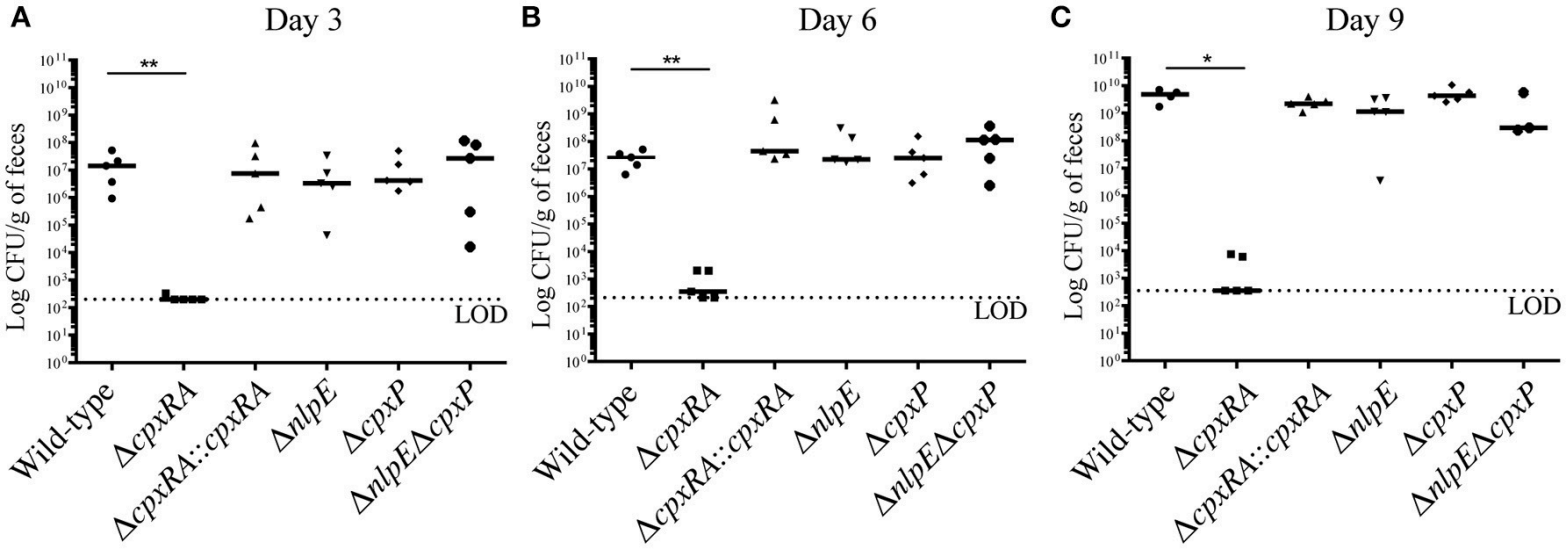
Bacterial burden in female C3H/HeJ mice after infection with Cpx TCS mutant strains of *Citrobacter rodentium*. Female C3H/HeJ mice were infected as described previously, and fecal bacterial burden was assessed at day 3 **(A)**, 6 **(B)**, and 9 **(C)** post infection by plating on MacConkey agar and counting Colony Forming Units (CFU). At day 9 post infection, due to significant illness manifestation, in the absence of fecal matter, colon was homogenized and plated on MacConkey agar. A Mann-Whitney test was used to determine significance between each mutant strain and wild-type (**p < 0.01; *p < 0.05). (LOD = Limit of detection; day 3, day 6: *n* = 5; day 9: *n* = 3–5; black bar denotes the median).

To assess the virulence contribution of the Cpx TCS auxiliary proteins (Figure 1A), we used *sacB* gene-based allelic exchange to generate knock out strains of *C. rodentium*, each lacking either NlpE (Δ*nlpE*), CpxP (Δ*cpxP*), or a double mutant (Δ*nlpE*Δ*cpxP*). We confirmed that both NlpE and CpxP are homologous to those found in *E. coli* K-12 MG1655, at 79 and 88% sequence identity, respectively. We infected susceptible C3H/HeJ mice with each strain, and virulence was assessed (Figures 1B, 2). As our group has previously reported, the Δ*cpxRA* deletion rendered the strain avirulent, with 100% survival of susceptible mice (Figure 1B) (Thomassin et al., 2015). As expected, the complemented Δ*cpxRA::cpxRA* strain restored virulence, with mortality comparable to wild-type (Figure 1B). The mice infected with the Cpx auxiliary protein mutant strains (Δ*nlpE*, Δ*cpxP*, Δ*nlpE*Δ*cpxP*) succumbed to infection with similar kinetics as the cohort infected with wild-type *C. rodentium* (Figure 1B). To further characterize this virulent phenotype, we assessed fecal *C. rodentium* loads at day 3 and 6 post infection, as well as colonic tissue *C. rodentium* loads at day 9 post infection. The wild-type *C. rodentium* loads increased as expected throughout infection, peaking at day 9 (Figure 2). As our group previously reported, the Δ*cpxRA C. rodentium* loads were significantly lower throughout the course of infection, an important internal control for our study (Figure 2) (Thomassin et al., 2015). Similarly as expected, the Δ*cpxRA::cpxRA* strain had *C. rodentium* loads comparable to wild-type levels at all time points (Figure 2) (Thomassin et al., 2015). The Cpx TCS auxiliary protein mutants, however, showed no significant difference in bacterial burden and the mice were colonized to levels similar to wild-type, at all time points (Figure 2). Taken together, these data indicate that the deletion of the reported sensors of the Cpx TCS (NlpE, CpxP) does not recapitulate the deletion of the TCS itself.

### Bacterial localization in the colon of susceptible mice infected with Cpx TCS auxiliary protein mutants is comparable to that of wild-type *C. rodentium*

**Figure 3:**
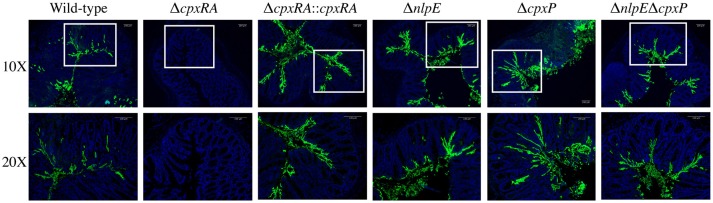
*In vivo* localization of wild-type and Cpx TCS mutant strains of *Citrobacter rodentium* in the intestine of C3H/HeJ mice. Localization of *C. rodentium* wild-type and Cpx TCS mutant strains in distal colon samples of day 9 infected C3H/HeJ mice. Sections stained with DAPI (blue) and anti-*Citrobacter* LPS (green). White boxes on 10X images denote the area of the 20X higher magnification image. Representative images shown from *n* = 3–4 biological replicates. Scale bars 100 μm.

We aimed to further characterize the implication of these sensors in *C. rodentium* virulence by localizing the bacteria in intestinal tissue of infected mice. Susceptible C3H/HeJ mice were infected as described in Materials and Methods, and euthanized on day 9 post infection. Colonic tissue sections were stained with DAPI, as well as anti - *C. rodentium* LPS antibody, in order to detect both the intestinal architecture and *C. rodentium* (Figure 3). Wild-type *C. rodentium* exhibited widespread localization on the colonic mucosal surface, with some localizing in the lumen of the intestine and some deeper within the crypts (Figure 3). Conversely, there was no detectable Δ*cpxRA C. rodentium* in the tissue sections, consistent with decreased bacterial loads (Figure 3). In contrast, the *cpxRA* complemented strain restored *C. rodentium* colonization and distribution to wild-type levels (Figure 3). Notably, the Δ*nlpE*, Δ*cpxP*, and Δ*nlpE*Δ*cpxP* strains all exhibited widespread colonization and localization across the mucosal surface, similar to wild-type (Figure 3).

### *In vitro* bacterial adherence of Cpx TCS and auxiliary protein mutants

**Figure 4:**
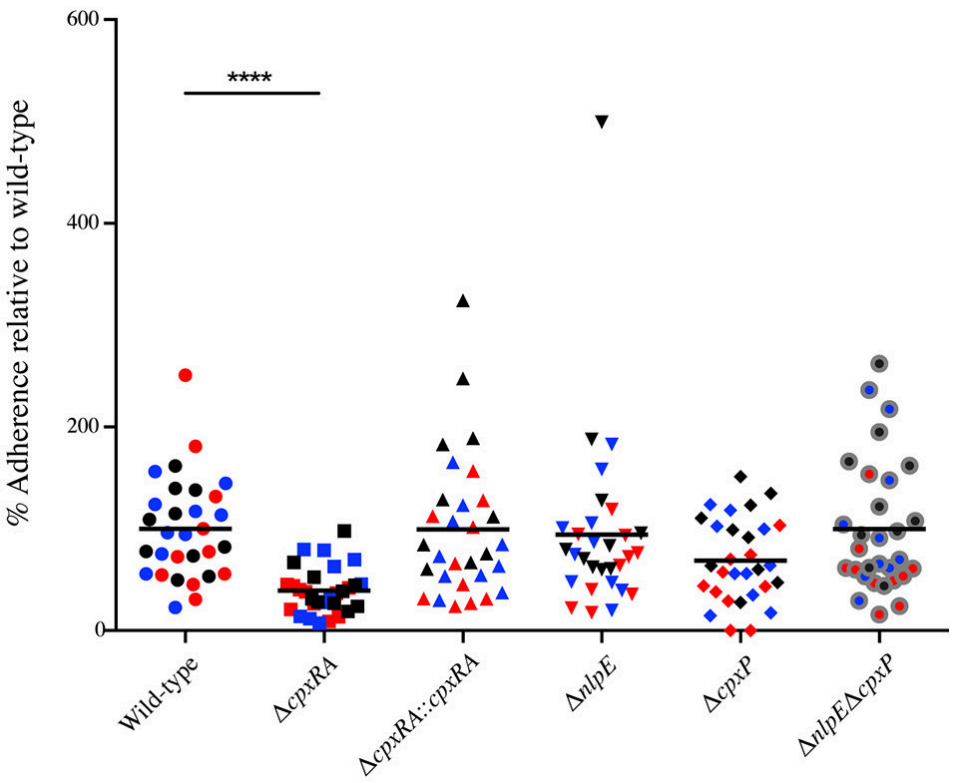
*In vitro* adherence of wild-type and Cpx TCS mutant strains of *Citrobacter rodentium* on HeLa cells. HeLa cells were infected with wild-type *C. rodentium* or a Cpx TCS mutant strain for 8 hrs. The samples were fixed and stained with DAPI and anti-*Citrobacter* LPS. Samples were imaged on a Zeiss Axiovert 200M microscope. Total number of bacteria per HeLa cell were counted. The 3 biological replicates for each strain are color-coded. Each replicate consists of 10 fields of view. A non-parametric one-way ANOVA was used to determine statistical significance (****P < 0.0001). Bars denote the mean.

Next, we wanted to further characterize the implication of these sensors in *C. rodentium* virulence by characterizing their ability to adhere to HeLa cells *in vitro*. HeLa cells were infected with wild-type or mutant *C. rodentium*, and then stained with DAPI and anti - *C. rodentium* LPS antibody, in order to enumerate total bacteria and cells. Consistent with the bacterial burden and *in vivo* localization data, the Δ*cpxRA* mutant displayed a significantly decreased ability (by 60%) to adhere to HeLa cells *in vitro* (Figure 4). The Δ*cpxRA::cpxRA* strain restored bacterial adherence to wild-type levels (Figure 4). Notably, the Cpx TCS auxiliary protein mutants Δ*nlpE*, Δ*cpxP*, and Δ*nlpE*Δ*cpxP* displayed adherence similar to wild-type, further supporting the fact that these mutants are fully virulent. As such, the *in vitro* adherence phenotypes presented in these assays further support the *in vivo* colonization phenotypes.

### General profile of gene expression between Δ*cpxra* and auxiliary protein mutants supports the respective virulence phenotypes

**Figure 5:**
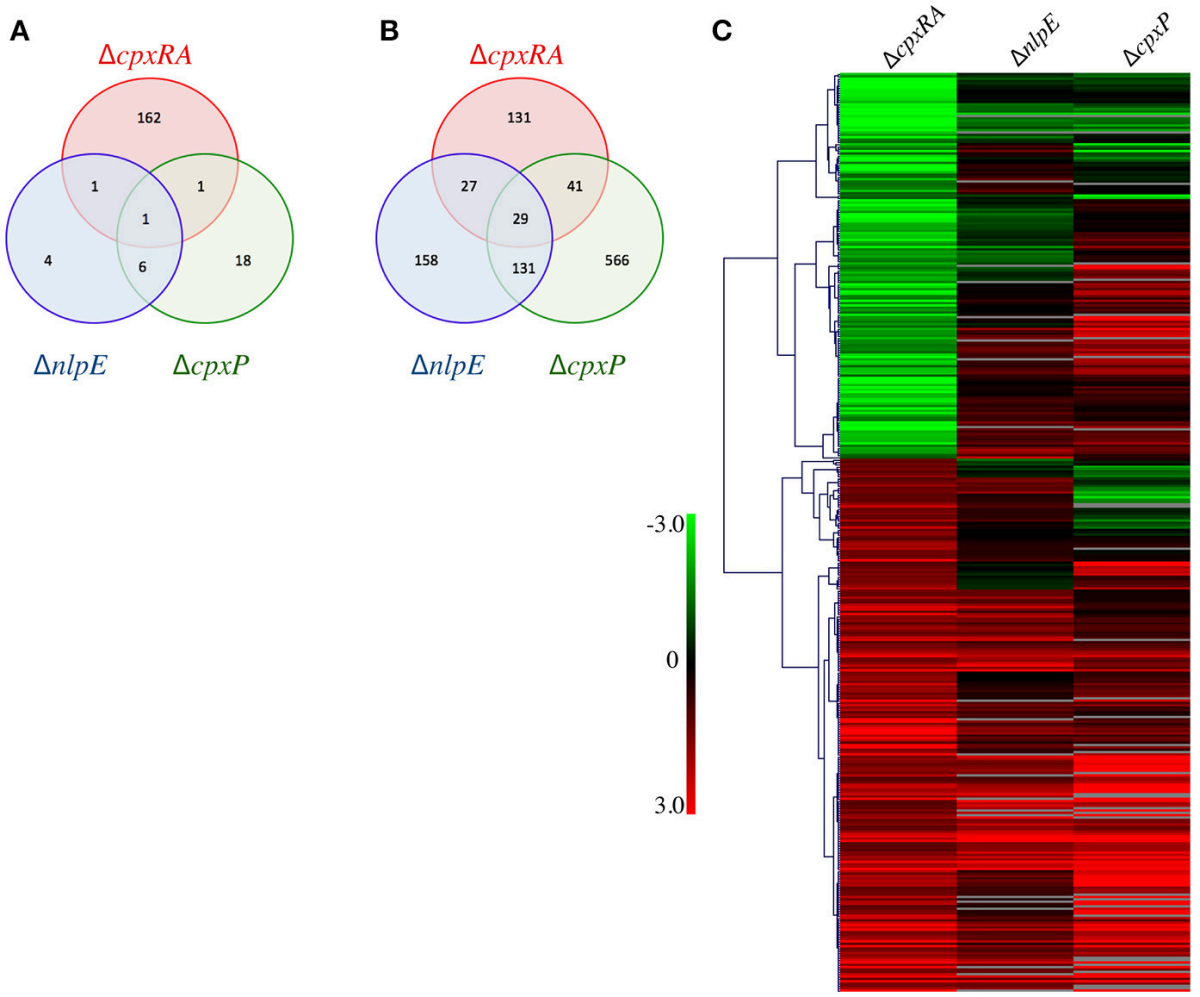
General gene expression profile of all mutant strains relative to wild-type. Strains were grown in DMEM and RNA was extracted from three biological replicates. The expression profiles of mutant strains were compared to that of the wild-type *C. rodentium*. **(A)** Venn diagram of the downregulated genes in each deletion strain. **(B)** Venn diagram of the upregulated genes in each deletion strain. **(C)** Genes differentially expressed in ΔcpxRA were clustered with Hierarchical clustering, using Pearson Uncentered correlation. Upregulated genes are shown in red, and downregulated genes are in green. Genes displayed are differentially expressed in the Δ*cpxRA C. rodentium* strain. An unpaired *t*-test was used to determine statistical significance (*p* < 0.05).

To further examine the disparity in phenotypes between our strains we sought to compare the regulons of the Cpx TCS and its auxiliary proteins. Our hypothesis is that genes repressed only in Δ*cpxRA*, but not in Δ*nlpE* and in Δ*cpxP* could be responsible for the virulence defect of Δ*cpxRA*. To this end, we performed microarray experiments on the wild-type, Δ*cpxRA*, Δ*nlpE*, and Δ*cpxP* strains of *C. rodentium* to determine the bacterial genes regulated by each TCS/sensor *in vitro*. We have previously confirmed that the expression profiles of the TCS during growth in DMEM closely resembles that seen *in vivo*, and hence used this condition to grow the strains (Thomassin et al., 2017). To gain insight into the virulence-associated CpxRA regulome, we focused our analysis on the genes differentially regulated (p <0.05, log_2_ ratio of mutant/wild-type >1 or <-1) in the Δ*cpxRA* strain compared to wild-type (Figure 5, Table S3). There are 393 genes differentially regulated in the Δ*cpxRA* strain relative to wild-type, of which 228 are upregulated and 165 genes are downregulated (Figures 5A,B). Further, there are 357 differentially regulated genes in the Δ*nlpE* regulon, of which 345 are upregulated and 12 are downregulated (Figures 5A,B). In the Δ*cpxP* regulon, there are 793 differentially regulated genes, of which 767 are upregulated and 26 are downregulated (Figures 5A,B). There exists some overlap in the genes differentially regulated in each of the single mutant strains, as seen in Figures 5A,B.

We hypothesized that genes downregulated in the Δ*cpxRA* strain, but either unchanged or upregulated in all other strains could be responsible for the virulence defect. To this end, we detected 162 genes significantly downregulated in the Δ*cpxRA* strain that were upregulated or unchanged in the Cpx auxiliary protein deletion strains. This list includes known virulence factors, outer membrane proteins, metabolism proteins, plasmids, and putative prophage genes (Table 1, Table S3).

**Table 1:** Selected genes significantly downregulated in Δ*cpxRA*.

**Function**	**Gene**	***T*-Test WT *vs*. Δ*cpxRA***	**Δ*cpxRA*-WT**	***T*-Test WT *vs*. Δ*nlpE***	**Δ*nlpE*-WT**	***T*-Test WT *vs*. Δ*cpxP***	**Δ*cpxP*-WT**
Biofilm regulator	*bssR*	0.0041	−2.54	0.2405	0.68	0.0217	1.96
Carbamoyl-phosphate synthase small chain	*carA*	0.0209	−1.26	0.4502	0.61	0.2437	−0.40
Cobalamin biosynthesis protein CbiG	*cbiG*	0.0211	−1.23	0.1584	0.41	0.0070	1.02
Colanic acid capsullar biosynthesis activation protein A	*rcsA*	0.0292	−3.45	0.3549	0.24	0.2449	−0.05
GntR-family transcriptional regulator		0.0054	−2.34	0.4119	1.15	0.0185	1.13
LysR-family transcriptional regulator	*ttdR*	0.0145	−1.67	0.2140	0.77	0.3299	−1.17
Maltodextrin phosphorylase	*malP*	0.0086	−4.75	0.0854	−1.52	0.3346	−1.27
Maltoporin (maltose-inducible porin)	*lamB*	0.0164	−5.91	0.1668	−2.38	0.1648	−2.55
Maltose operon periplasmic protein	*malM*	0.0042	−4.39	0.0647	−1.17	0.2268	−1.30
Maltose transport system, permease protein	*mslF*	0.0089	−5.80	0.2379	−1.34	0.4134	0.06
Maltose transport system, permease protein	*malG*	0.0068	−5.48	0.2072	−1.30	0.1935	−1.97
Maltose transport system, substrate-binding protein	*malE*	0.0126	−5.88	0.2793	−1.76	0.1719	−1.99
Maltose/maltodextrin transport system,ATP-binding protein	*malK*	0.0116	−4.27	0.2263	−1.80	0.2833	1.74
Mannose-specific PTS system EIIAB component	*manX*	0.0043	−1.76	0.3941	0.18	0.0856	1.04
Mannose-specific PTS system EIIAB component	*manX*	0.0084	−1.52	0.4406	−0.51	0.0397	0.68
Outer membrane protein F	*ompF*	0.0009	−4.29	0.3480	0.33	0.1720	1.02
Protein Bdm (biofilm-dependent modulation protein)	*bdm*	0.0443	−3.36	0.4975	1.44	0.0619	1.16
Putative colanic acid biosynthesis glycosyl transferase	*wcaL*	0.0025	−1.61	0.3977	0.59	0.1976	2.80
Putative lipoprotein	*ygdI*	0.0029	−2.19	0.1360	0.84	0.0114	1.28
Putative T3SS effector protein EspO	*espO*	0.0038	−2.87	0.1631	−0.83	0.4596	0.03
Universal stress protein F	*uspF*	0.0215	−2.64	0.3482	0.21	0.1871	0.54
VgrG family T6SS protein Cts1G OR T6SS protein Cts1F	*cts1G, cts1F*	0.0203	−1.77	0.2643	0.80	0.2062	2.22

Genes differentially expressed in Δ*cpxRA* were analyzed with Hierarchical clustering, using the Pearson Uncentered correlation (Figure 5C). For some genes, the values for Δ*cpxP*, and Δ*nlpE* are not statistically significant. See Table S3 for details. As seen in Figure 5C, the expression profile of the Δ*nlpE* strain correlates well with patterns seen in the Δ*cpxRA* strain for genes repressed by CpxRA (upregulated in the mutant). However, in the subset of genes activated by CpxRA, the correlation between the Δ*nlpE* and Δ*cpxRA* strains is not evident, indicating that in these conditions, induction of gene expression by CpxRA is acting in a largely NlpE-independent manner. If NlpE was required for activation of the Cpx TCS, all genes downregulated in the Δ*cpxRA* strain would also be downregulated in the Δ*nlpE* strain. Instead, we found that the majority of these genes were upregulated or unchanged (Figure 5). Further, since CpxP is proposed to be a negative regulator of the Cpx TCS, we hypothesized that gene expression in Δ*cpxRA* would be inversely correlated with the gene expression in the Δ*cpxP* strain. To the contrary, we detect a correlation similar to that of the Δ*nlpE* strain, where the repressed genes correlate with expression patterns in the Δ*cpxP* strain, and only some of the activated genes are inversely correlated, suggesting that CpxP is not acting as a global negative regulator of the Cpx response in these conditions (Figure 5).

## Discussion

In the dynamic setting of infection, bacteria must be able to sense environmental cues and adapt gene expression accordingly. Through the use of two-component systems (TCSs), bacteria utilize an inner membrane-bound sensor kinase to sense a signal, which is subsequently transferred to a cytoplasmic response regulator through phosphate transfer (Mascher et al., 2006). The cytoplasmic response regulator binds to cognate DNA sequences in specific target genes in order to alter their expression (Mascher et al., 2006). Several TCSs have been implicated in the virulence of A/E pathogens, both by our group and others (Thomassin et al., 2015, 2017; Moreira et al., 2016). In this study, we specifically evaluate the CpxRA TCS virulence defect in the context of upstream signaling through the NlpE lipoprotein. We also aim to determine the effect of the negative regulator protein CpxP on virulence. The Δ*nlpE* and Δ*cpxP* strains exhibited no defect in virulence, as the mice succumbed to infection with similar kinetics to those infected with wild-type *C. rodentium*. Further, *in vivo* localization experiments and *in vitro* adherence experiments showed no difference between the auxiliary protein mutants and wild-type. As such, we suggest that the virulence defect previously shown in the absence of CpxRA is independent of both NlpE and CpxP.

With the use of microarrays, we determined the regulon of the Δ*cpxRA*, Δ*nlpE*, and Δ*cpxP* deletion strains. Based on their proposed functions, we hypothesized that genes differentially expressed in the Δ*cpxRA* strain should be directly correlated with those in the Δ*nlpE* strain, and inversely correlated with those in the Δ*cpxP* strain. In contrast to our hypothesis, we found that a significant number of genes in the Δ*cpxRA* deletion strain are regulated independently of both auxiliary proteins. As such, the genes regulated by CpxRA independently of NlpE and CpxP are good candidates to explain the lack of virulence of the Δ*cpxRA* strain. We hypothesize that the virulence-associated effect of CpxRA is due to the differential regulation of a combination of genes, rather than a single gene. Certain candidate genes, however, are likely to play a more significant role than others.

The Type 3 Secretion System (T3SS) is absolutely essential to the virulence of A/E pathogens. As such, screening for the downregulation of effectors and translocators of this system is an attractive target for virulence defects. We detected an uncharacterized putative T3SS effector, annotated as *espO*, to be downregulated in the Δ*cpxRA* strain, but unchanged in all other strains, relative to wild-type *C. rodentium* (Table 1, Table S3).

Another candidate that arose in our study is the Type VI Secretion System (T6SS), present in Gram-negative bacteria. The T6SS is a membrane-spanning molecular structure which is assembled in the cytoplasm before being propelled toward a target bacterial or eukaryotic cell (Zoued et al., 2014). The T6SS transfers effectors to the target cell in order to cause cell damage. It can inject toxins into eukaryotic cells that interfere with the cytoskeleton, and also translocate antibacterial effectors targeting bacterial cells directly (Pukatzki et al., 2007; Russell et al., 2011; Zoued et al., 2014). The genome of *C. rodentium* harbors two T6SS clusters, CTS1 and CTS2 (Petty et al., 2010; Gueguen and Cascales, 2013). CTS1 has a frameshift mutation in the *cts1I* gene which results in a premature stop codon (Gueguen and Cascales, 2013). However, due to a sequence of consecutive adenosines prior to this stop codon, slippage can occur, leading to a functional CTS1 T6SS (Gueguen et al., 2014). Two genes in the CTS1 cluster, *cts1G* and *cts1F*, are downregulated in the Δ*cpxRA* regulon (Table 1). The *cts1F* gene is a Forkhead-associated protein, and *cts1G* is associated with the VgrG family (Petty et al., 2010). VgrG is exposed at the surface and acts as a cell-puncturing device, before likely being secreted into the host cell, where is has been reported to crosslink actin *in vitro* (Pukatzki et al., 2007). As such, a downregulation in this system could limit the ability of *C. rodentium* to compete with commensal bacteria for access to the intestinal epithelium, which is crucial for its ability to colonize the host or interact with eukaryotic cells. This effect would possibly make *C. rodentium* unfit for colonization and survival within the gut.

Maltose is essential for the colonization of the gut by EHEC O157:H7 (Jones et al., 2008). We detect the downregulation of multiple genes involved in maltose metabolism in the Δ*cpxRA* strain, whereas they are unchanged in the auxiliary protein mutants. These genes include the maltoporin *lamB*, the substrate-binding protein *malE*, the permease protein *malG*, the maltodextrin phosphorylase *malP*, the periplasmic protein *malM*, the ATP-binding protein *malK*, and the permease protein *mslF* (Table 1, Table S3). These genes are involved in maltose and maltodextrin transport and metabolism in commensal and pathogenic *E. coli* (Jones et al., 2008). Further, maltose metabolism genes are upregulated in *E. coli* in the presence of mucus, suggesting a role in the gut environment (Chang et al., 2004). As such, the downregulation of these genes could render the Δ*cpxRA* strain unable to use maltose as a nutrient source, which may be important in the intestine.

We detected a significant downregulation of the gene *ygdI*, in the Δ*cpxRA* strain, while remaining unchanged in the Δ*nlpE* strain and upregulated in the Δ*cpxP* strain. *In silico* analysis using CD-search, TMHMM and SignalP revealed that YgdI is a putative small lipoprotein with a DUF903 domain, a Sec-dependent signal sequence, and a putative transmembrane helix which is likely cleaved during export (Krogh et al., 2001; Petersen et al., 2011; Marchler-Bauer et al., 2017). Taken together, this *in silico* analysis suggests that the YgdI lipoprotein may be localized to the periplasm. Further, YgdI is highly and positively regulated by RpoS, a sigma factor that responds to a multitude of stressors (Dong and Schellhorn, 2009; Saint-Ruf et al., 2014). Since a significant role of the Cpx TCS is to respond to misfolded proteins (Snyder and Silhavy, 1995), it is conceivable that YgdI could be involved in mitigating membrane or periplasmic stress resulting in misfolded proteins. This will require further experimentation.

Further, two biofilm-related genes were downregulated in Δ*cpxRA*. *bdm*, a biofilm-dependent modulation gene, was repressed 10-fold relative to the wild-type (Table 1, Table S3). *bdm* expression is known to be reduced within biofilms; however, overexpression reportedly increased biofilm production (Prigent-Combaret et al., 1999; Sim et al., 2010). *bdm* expression occurs in response to osmotic shock and is reported to be under the control of the Rcs TCS, thereby suggesting a link between the Rcs and Cpx TCSs (Francez-Charlot et al., 2005). *bssR*, a biofilm regulator, was downregulated approximately 6-fold in Δ*cpxRA*. The *bssR* transcript is induced in the stationary phase and has been shown to impact biofilm formation through a complex pathway involving indole regulation, and the uptake and export of autoinducer 2 (Selinger et al., 2000; Domka et al., 2006). While the link between biofilm formation and virulence is ill-defined in *C. rodentium*, overlap between these two systems is documented in other bacterial species and is mediated by quorum sensing systems (Cotter and Stibitz, 2007).

The Δ*cpxRA* virulence defect may be due to the strain's inability to adhere adequately to epithelial cells. Our *in vitro* adherence experiments uncovered a striking decrease in adherence in this strain, similar to what we see in our *in vivo* localization experiments. We detected a slight upregulation in some genes of the classical *C. rodentium* type 4 pilus operons, *kfc* and *cfc*, in multiple strains, including Δ*cpxRA* (Table S3) (Mundy et al., 2003; Hart et al., 2008). These operons are largely associated with intestinal colonization and adherence; however, there was no pattern of expression among the strains which could explain the virulence phenotypes. Nevertheless, we detect significant downregulation of a number of genes which could be indirectly contributing to adherence through the alteration of the outer membrane or the capsule of the bacterium. Namely, we detected decreased levels of *ompF*, which makes up a large component of the outer membrane. While previous work in *E. coli* uncovered that the Cpx TCS negatively controls expression of *ompF*, we found that loss of *cpxRA* lead to the downregulation *of ompF* (Batchelor et al., 2005). In avian pathogenic *E. coli* the loss of *ompF* significantly decreases bacterial virulence in both duck and mouse models of infection, as well as adherence *in vitro* (Rolhion et al., 2007; Hejair et al., 2017). We also detected a downregulation of the universal stress protein F, *uspF*, in Δ*cpxRA*, compared to unchanged expression levels in all other strains. Universal stress proteins compose a family of proteins which respond to different types of cellular stress, including oxidative stress (Kvint et al., 2003). In *E. coli, uspF*, a member of the second class of these proteins, promotes fimbria-mediated adhesion of the bacteria (Nachin et al., 2005). In atypical EPEC, *uspF* expression was detected at high levels in response to oxidative stress, low pH, high salt concentration, and heat (de Souza et al., 2016). The decreased expression of *uspF* in Δ*cpxRA* could render the strain unfit to combat cellular stress and/or adhere to the epithelium. Another gene uncovered in our studies is the colanic acid capsular biosynthesis activation protein A, or *rcsA*, which is part of the Rcs phosphorelay TCS, as well as *wcaL*, a putative colanic acid biosynthesis glycosyl transferase (Thomassin et al., 2017). Colanic acid is a capsule protein, which could alter the bacterium's ability to adhere to surfaces. We have previously shown that the deletion of the Rcs TCS leads to moderate attenuation of *C. rodentium*, suggesting that the lack of virulence in the Δ*cpxRA* strain could be the result of crosstalk between the Cpx and Rcs TCSs (Thomassin et al., 2017). However, the attenuation of the Rcs TCS mutant was much less pronounced than that of the CpxRA mutant, indicating that decreased expression of *rcs* on its own is unlikely to account for the attenuation of the Δ*cpxRA* mutant.

We also detected multiple significantly downregulated transcriptional regulators in the Δ*cpxRA* strain, which are either upregulated or unchanged in the other strains (Table 1, Table S3). Specifically, we detected the transcriptional regulators, *yiaG*, and *ttdR*, suggesting that the virulence defect could be regulated indirectly by CpxRA (Table 1).

In conclusion, this study characterizes the *Citrobacter rodentium* virulence defect caused by the deletion of the CpxRA TCS to be independent of NlpE and CpxP. The Δ*nlpE* and Δ*cpxP* deletion strains were fully virulent and able to adhere to cells both *in vitro* and *in vivo*. Furthermore, we studied the regulon of each of these strains in an effort to uncover the gene(s) responsible for this effect. Future studies will aim to uncover the exact mechanism of attenuation of the Cpx TCS deletion strain, which is likely to involve a multitude of factors, such as T6SS, *ompF, uspF*, and colanic acid. The delineation of this mechanism could uncover future therapeutic targets for the treatment of enteric pathogens.

## Author contributions

NG, NM, and LZ participated in the design of the experiments and performed the experiments. HLM, SG, and SF designed the experiments and supervised the work. All authors participated in data analysis. The manuscript was written by NG and edited by NM, HLM, SG, and SF.

### Conflict of interest statement

The authors declare that the research was conducted in the absence of any commercial or financial relationships that could be construed as a potential conflict of interest.
