# Supplementary material for: The Virulence Effect of CpxRA in Citrobacter rodentium Is Independent of the Auxiliary Proteins NlpE and CpxP
Source: Front Cell Infect Microbiol. 2018 Sep 18;8:320. doi: 10.3389/fcimb.2018.00320 (PMC6153362; doi:10.3389/fcimb.2018.00320)
Supplement: Supplementary file 1 [file Table_1.docx]

**Table S1: Strains and plasmids used in this study**

| **Strain or plasmid** | **Description** | **Reference or source** |
| --- | --- | --- |
| *Citrobacter rodentium* |  |  |
| Wild-type | *C. rodentium* DBS100 | (Schauer and Falkow, 1993) |
| *ΔcpxRA* | DBS100 *ΔcpxRA* | (Thomassin et al., 2015) |
| *ΔcpxRA::cpxRA* | DBS100 *ΔcpxRA::cpxRA* | (Thomassin et al., 2015) |
| *ΔnlpE* | DBS100 *ΔnlpE* | This study |
| *ΔcpxP* | DBS100 *ΔcpxP* | This study |
| *ΔnlpEΔcpxP* | DBS100 *ΔnlpEΔcpxP* | This study |
| *Escherichia coli* |  |  |
| χ7213 | *thr-1 leuB6 fhuA21 lacY1 glnV44 recA1 asdA4 thi-1* RP4-2-Tc::Mu [- *pir*] Kanr | (Roland et al., 1999) |
| χ7213(pΔ*nlpE*) | χ7213 containing pΔ*nlpE* | This study |
| χ7213(pΔ*cpxP*) | χ7213 containing pΔ*cpxP* | This study |
| **Plasmids** |  |  |
| pRE112 | Sucrose-sensitive (*sacB1*) suicide vector; Cmr | (Edwards et al., 1998) |
| p*ΔnlpE* | *nlpE* deletion construct in pRE112 | This study |
| p*ΔcpxP* | *cpxP* deletion construct in pRE112 | This study |
