# Supplementary material for: The Virulence Effect of CpxRA in Citrobacter rodentium Is Independent of the Auxiliary Proteins NlpE and CpxP
Source: Front Cell Infect Microbiol. 2018 Sep 18;8:320. doi: 10.3389/fcimb.2018.00320 (PMC6153362; doi:10.3389/fcimb.2018.00320)
Supplement: Supplementary file 2 [file Table_2.docx]

**Table S2: Primers used to generate deletion strains**

| **Deletion strain** | **Primer** | **Sequence (5’ → 3’)** | **Restriction site** |
| --- | --- | --- | --- |
| Δ*nlpE* | nlpE_1 | GCTCTAGAGGTTGTTAATGTGGCGGATCTCG | XbaI |
|  | nlpE_2 | CCGCTCGAGTATCGCGGAGATGAGTGTCTTT | XhoI |
|  | nlpE_3 | CCGCTCGAGCCAGGACTGTAACAGTAAATAA | XhoI |
|  | nlpE_4 | GGGGTACCTTGTCGAAGGTCGTGCCTAAA | KpnI |
| Δ*cpxP* | cpxP_1 | GCTCTAGAGGCTATTATCTGGCCGTAGTATAA | XbaI |
|  | cpxP_2 | CCGCTCGAGTAGCAACTCACGTTCCCAGTAA | XhoI |
|  | cpxP_3 | CCGCTCGAGGCAGCGGTAACTTTGCGCAT | XhoI |
|  | cpxP_4 | GGGGTACCGAGTCGAGCTTGGGCAACATCA | KpnI |
